# Higher risk for cervical herniated intervertebral disc in physicians: A retrospective nationwide population-based cohort study with claims analysis

**DOI:** 10.1097/MD.0000000000005055

**Published:** 2016-10-14

**Authors:** Cheng Liu, Chien-Cheng Huang, Chien-Chin Hsu, Hung-Jung Lin, How-Ran Guo, Shih-Bin Su, Jhi-Joung Wang, Shih-Feng Weng

**Affiliations:** a Hyperbaric Oxygen Therapy Center and Division of Plastic Surgery, Chi-Mei Medical Center; b Department of Electrical Engineering, Southern Taiwan University of Science and Technology; c Department of Emergency Medicine, Chi-Mei Medical Center; d Department of Environmental and Occupational Health, College of Medicine, National Cheng Kung University; e Bachelor Program of Senior Service, Southern Taiwan University of Science and Technology; f Department of Occupational Medicine; g Department of Geriatrics and Gerontology, Chi-Mei Medical Center; h Department of Biotechnology, Southern Taiwan University of Science and Technology, Tainan; i Department of Emergency Medicine, Taipei Medical University, Taipei; j Department of Occupational and Environmental Medicine, National Cheng Kung University Hospital; k Department of Leisure, Recreation and Tourism Management, Southern Taiwan University of Science and Technology; l Department of Medical Research, Chi-Mei Medical Center, Liouying; m Department of Medical Research, Chi-Mei Medical Center, Tainan; n Department of Healthcare Administration and Medical Informatics, Kaohsiung Medical University, Kaohsiung, Taiwan.

**Keywords:** cervical, herniated intervertebral disc, physician

## Abstract

There is no study about cervical herniated intervertebral disc (cervical HIVD) in physicians in the literature; therefore, we conceived a retrospective nationwide, population-based cohort study to elucidate the topic. We identified 26,038 physicians, 33,057 non-physician healthcare providers (HCPs), and identical numbers of non-HCP references (i.e., general population). All cohorts matched a 1:1 ratio with age and gender, and each were chosen from the Taiwan National Health Insurance Research Database (NHIRD). We compared cervical HIVD risk among physicians, nonphysician HCPs, and non-HCP references and performed a follow-up between 2007 and 2011. We also made comparisons among physician specialists. Both physicians and nonphysician HCPs had higher cervical HIVD risk than non-HCP references (odds ratio [OR]: 1.356; 95% confidence interval (CI): 1.162–1.582; OR: 1.383; 95% CI: 1.191–1.605, respectively). There was no significant difference of cervical HIVD risk between physicians and nonphysician HCPs. In the comparison among physician specialists, orthopedists had a higher cervical HIVD risk than other specialists, but the difference was not statistically significant (adjusted OR: 1.547; 95% CI: 0.782–3.061). Physicians are at higher cervical HIVD risk than the general population. Because unknown confounders could exist, further prospective studies are needed to identify possible causation.

## 1 Introduction

Cervical herniated intervertebral disc (HIVD) is a common cause of pain and numbness in the neck, shoulder, or arm, which may radiate to the hands or fingers, and often resembles other musculoskeletal diseases, including carpal tunnel syndrome and rotator cuff syndrome.^[1]^ Other symptoms such as headache, dizziness/vertigo, and motor weakness may also exist.^[2]^ These symptoms cause affected patients much suffering and may disable them. The common risk factors are age, lack of regular exercise, tobacco use, poor posture (i.e., incorrect lifting or twisting causing additional stress on the cervical spine), and injury.^[1]^

Physicians in Taiwan work long hours and face much stress, particularly since the launch of National Health Insurance (NHI) in 1995, which provides 99.9% coverage of Taiwan's population, offering cheap and easy access to health care.^[3–5]^ Long working hours may lead to a lack of regular exercise and poor posture (e.g., standing for operation), which are risk factors for cervical HIVD. Using “cervical,” “herniated intervertebral disc,” and “physicians” as key words to search for literature indexed in PubMed and Google Scholar, we did not find any reports on this topic. Therefore, we conceived a retrospective nationwide population-based cohort study with claims analysis to clarify the comparison of risk for cervical HIVD between physicians and the general population. Because there are no data about work hours and other risk factors in the database we used, we focused the analyses on the comparisons of different populations defined by occupation.

## 2 Methods

### 2.1 Data source

For analysis, we used the 2009 Registry for medical personnel (PER) and the Longitudinal Health Insurance Database 2000 (LHID2000), 2 subsets of the NHI Research Database (NHIRD), which contains registration files and original claim data for reimbursement.^[6]^ Large, computerized databases derived from this system by the NHI Administration, Ministry of Health and Welfare, Taiwan, and maintained by the National Health Research Institutes, Taiwan, are provided to scientists in Taiwan for research purposes.^[6]^ The LHID2000 contains all the original claim data of 200,000 individuals randomly sampled from the 2000 Registry for Beneficiaries of the NHIRD, which maintains the registration data of every beneficiary of the NHI program during the period 1996–2000.^[7]^ All registration and claim data of the nearly 1,000,000 individuals collected by the NHI program constitutes the LHID2000. There is no significant difference in gender distribution.^[7]^ The 2009 Registry for medical personnel (PER) contains the information on all healthcare providers (HCPs) registered in 2009 including physicians, nurses, pharmacists, and other HCPs. It includes types of HCPs, physician specialty, date licensed, work area, hospital level, types of employment, and encrypted identification number, which can be linked to the aforementioned claim data in NHIRD. By the linking, we can compare between HCPs and general population.

### 2.2 Study and reference cohorts

**Figure 1:**
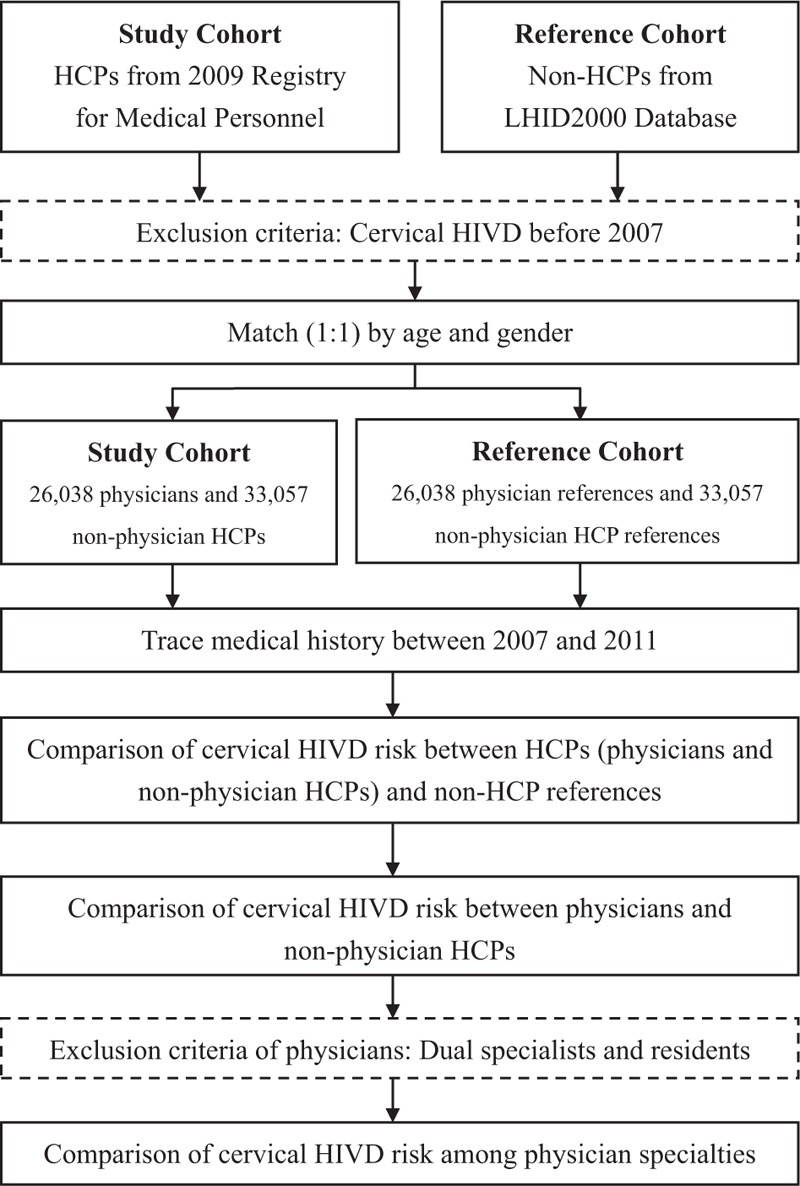
Flowchart of study. HCP = health care provider, HIVD = herniated intervertebral disc, LHID = longitudinal Health Insurance Database.

We identified 26,038 physicians and 33,057 non-physician healthcare providers (HCPs) as the study cohort from the 2009 PER, and an equal number of non-HCP references (general population), a total of 59,095, from the LHID2000 (Fig. 1). We identified nonphysician HCPs to compare with physicians to eliminate a possible “healthy worker effect”^[8]^ and to evaluate whether physicians have a greater possibility for cervical HIVD than their coworkers. The identified nonphysician HCPs were audiologists, consultant experts, clinical experts, dietitians, social workers, and language experts. We excluded participants who had been treated for cervical HIVD before 2007. Matching between physicians and non-HCP references and between nonphysician HCPs and non-HCP references was performed by a 1:1 ratio with age and gender (Fig. 1). We matched age between HCPs and non-HCP references because it is a major risk factor for cervical HIVD.^[1]^ We also matched gender because the gender ratio is very different between HCPs and non-HCP references, which may confound the analysis. We applied a Statistical Analysis System macro (gmatch), which used a greedy-matching algorithm to select the nearest reference for each HCP without replacement. The nearest reference was selected by using weighted sum of the difference between HCPs and references.

### 2.3 Comparison of cervical HIVD risk between HCPs and non-HCP references, between physicians and nonphysician HCPs, and among physician specialists

First, we compared cervical HIVD (ICD-9 code: 7220, 7224, 72271, and 72291) risk among HCPs (physicians and nonphysician HCPs) and non-HCP references by tracing their medical histories between 2007 and 2011. Second, we compared cervical HIVD risk between physicians and nonphysician HCPs by similar means. Third, we compared cervical HIVD risks among physician specialists. Our physician specialists worked in the fields of internal medicine, pediatrics, ob/gyn, emergency medicine, orthopedics, surgery, and other specialties. Physicians with dual-specialist roles and residents were excluded due to the difficulty of dividing specific specialties and short working history, respectively.

### 2.4 Ethic statements

This study was conceived according to the Declaration of Helsinki and approved by the Institutional Review Board at Chi-Mei Medical Center. Because the data contained only nonidentity information, informed consent was waived for the participants. The welfare and rights of participants were not affected by the waiver.

### 2.5 Statistical analyses

We used SAS 9.3.1 for Windows (SAS Institute, Cary, NC) for all analyses. Significance was set at *P* < 0.05 (2-tailed). In the comparison of demographic characteristics and comorbidities between HCPs (physicians and non-physician HCPs) and non-HCP references, we used the independent *t*-test for continuous variables and chi-square for categorical variables. We compared cervical HIVD risk between HCPs (physicians and nonphysician) and non-HCP references (general population) by conditional logistic regression. A comparison between physicians and nonphysician HCPs was made by unconditional logistic regression. Then, we compared cervical HIVD risk among physician specialists, using “other specialists” as the reference, by unconditional logistic regression.

## 3 Results

The mean age and male ratio between physicians and nonphysician HCPs was 46.55 ± 10.78 years vs 34.57 ± 7.65 years and 85.19% vs 36.77%, respectively (Table 1). Physicians had significantly higher cervical HIVD risk than non-HCP references (odds ratio [OR]: 1.356; 95% confidence interval [CI]: 1.162–1.582) (Table 2). Nonphysician HCPs also had significantly higher cervical HIVD risk than non-HCP references (OR: 1.383; 95% CI: 1.191–1.605).

**Table 1:**
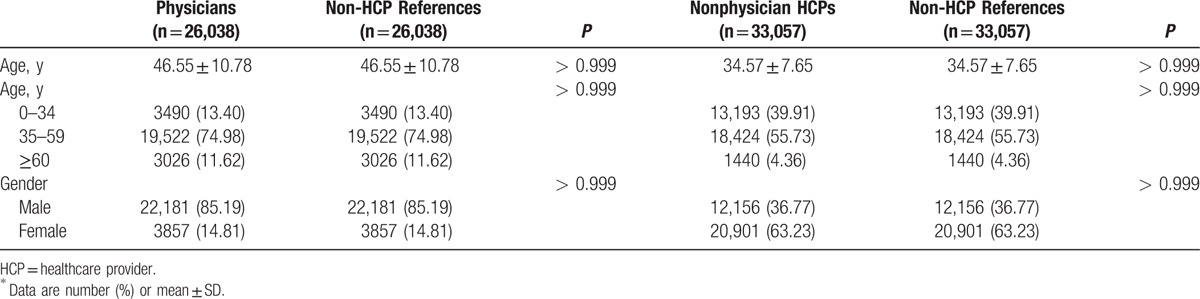
Demographic characteristics and comorbidities for physicians, nonphysician HCPs, and non-HCP references (general population)^∗^.

**Table 2:**
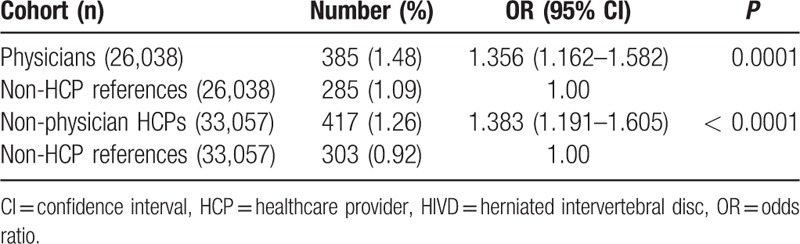
Comparison of risk for cervical HIVD between HCPs (physicians and nonphysicians) and non-HCP references (general population) by conditional logistic regression.

In the comparison between physicians and nonphysician HCPs, physicians had a higher prevalence of cervical HIVD (1.48% vs 1.26%); however, it was not statistically significant (adjusted OR [AOR]: 1.063; 95% CI: 0.904–1.250) (Table 3). A similar result occurred in the stratification analysis of gender.

**Table 3:**
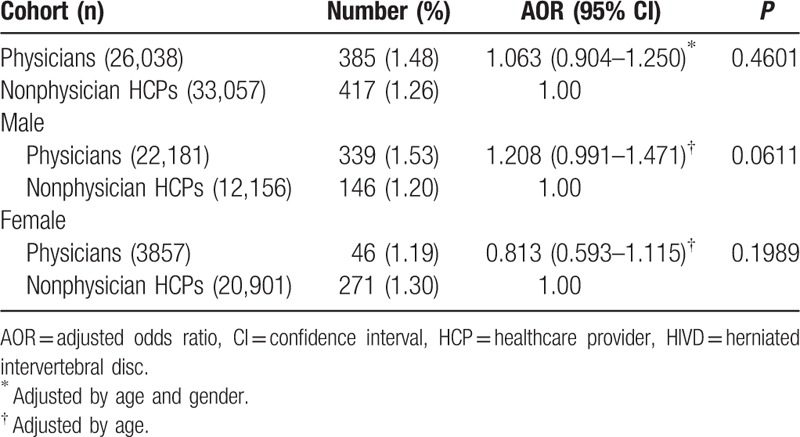
Comparison of risk for cervical HIVD between physicians and non-physician HCPs by unconditional logistic regression.

In the comparison among physician specialists, orthopedists trended for higher cervical HIVD risk than other specialists but was not statistically significant (AOR: 1.547; 95% CI: 0.782–3.061) (Table 4).

**Table 4:**
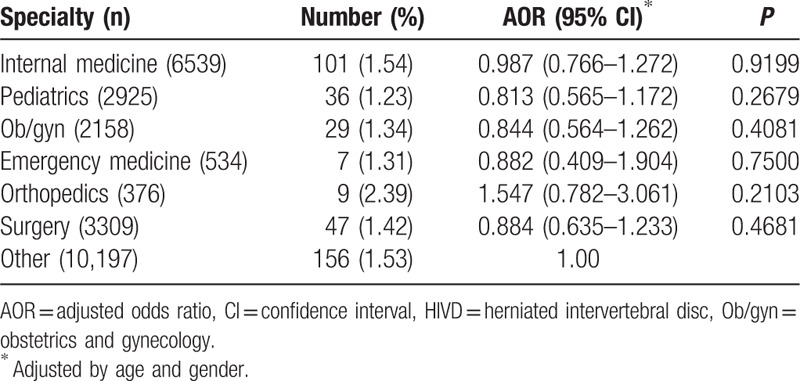
Comparison of risk for cervical HIVD among physician specialists by unconditional logistic regression.

## 4 Discussion

This retrospective nationwide population-based cohort study delineated that physicians have a higher cervical HIVD risk than the general population. There was no significant difference of cervical HIVD risk between physicians and nonphysician HCPs. Orthopedists tended to have a higher cervical HIVD risk than other specialists, but the increased risk was not statistically significant. Researchers should conduct future prospective studies that would identify possible causation.

Neck pain is a disturbing problem, affecting ∼10% of the adult population^[9]^; it has a similar prevalence to low back pain. Cervical HIVD is a common cause of neck pain and cervical radiculopathy, which is caused by the combination of intervertebral pressure and degeneration of the ligamentous fibers. This leads to a tear in the annulus, allowing the nucleus pulposus to prolapse through the annulus.^[2]^ An epidemiologic study of cervical radiculopathy^[10]^ reported that the mean age at diagnosis was 47.9 years; the male to female ratio was 1.7. The most affected area, ∼70% of patients, was the lower cervical spine, particularly C7.^[11]^ Many risk factors are responsible for cervical HIVD: aging; poor lifestyle; including tobacco use, lack of regular exercise, and inadequate nutrition; and poor posture such as standing for a long time, sitting, and incorrect lifting or twisting.^[2]^

Longer working hours may contribute to a poor posture and lifestyle,^[1]^ which are risk factors for the development of cervical HIVD in physicians. Despite no research on cervical HIVD in physicians, previous studies have reported that occupations involving heavy manual labor such as meat carriers, dentists, miners, and drivers, leading to frequent extreme positions of the cervical spine pose as a risk of cervical HIVD.^[12–14]^ Because the availability of NHI has increased the demand for healthcare in Taiwan, the number of outpatient visits per person increased from 7.89 in 1992 to 15.2 in 2010.^[4,15,16]^ The annual number of outpatient visits per physician in Taiwan was only 6621 in 1992; it has increased by nearly 30% to 8600 in 2012.^[4,15,16]^ Both long time sitting in outpatient clinics and standing in operating rooms may predispose physicians to cervical HIVD.

Age-related degeneration cannot be changed; however, in addition to decreasing workload, other strategies, including avoiding poor posture, enhancing exercise, and ceasing tobacco use, are probable ways to prevent cervical HIVD in physicians.^[1,2,12–14]^ Cervical HIVD is a work-related musculoskeletal disease (WMSD), which is the most common occupational disease in the world.^[17]^ The common contributing factors for WMSDs are repetitiveness of work, applied force, fixed body positions, and the pace of work.^[17]^ Ways to prevent WNSDs include mechanization, job rotation, job enlargement, enrichment and teamwork, workplace design, and tools and equipment design.^[17]^ These may also be useful strategies for preventing cervical HIVD.

Although this study was the first nationwide, population-based cohort study delineating a pilot result, there were some limitations. First, there was no detailed information in accessed databases regarding lifestyles, tobacco use, exercise, nutrition, posture, and history of injury, which may be confounding factors for cervical HIVD. Second, NHIRD did not have the data on work hours and other risk factors, and therefore, we could not evaluate the correlations of cervical HIVD with work hours and other risk factors directly. For example, we cannot confirm that the physicians experiencing long work hours, per se, are the physicians with disease. What if those physicians with disease actually work less hours, but merely do not exercise, and so on. What if they acquired the disease as students with undergraduate years of poor posture. Possibly other factors or a new, as yet unknown, factor may be the cause. Third, physicians may choose to self-treat when they have diseases,^[18,19]^ and therefore we might underestimate the risk in physicians by using the claim data. Since we observed an increased risk in physicians in comparison with the general population, this limitation would not change our main conclusion. Nonetheless, this might contribute to the comparisons among the physician specialists. For example, if specialists of orthopedics, who had the highest risk of developing cervical HIVD among physicians, were more likely to self-treat themselves than other specialists, this limitation would have lead to an underestimation of the risk, making the risk not statistically significant. Fourth, the NHIRD uses ICD-9 for coding, but better coding instruments such as ICD-10 may be better aid in understanding causation of disease processes. Fifth, the follow-up period (2007–2011) may not be adequate, and a longer follow-up study may be needed to augment the results. Sixth, in spite of its nationwide nature, the present study's results may not be generalized to other nations due to the differing workloads of physicians in different countries.

## 5 Conclusions

This is the first study delineating the risk of cervical HIVD between physicians and the general population. Physicians had more cervical HIVD risk than the general population. Orthopedists trended higher in cervical HIVD risk than other specialists; however, it was not statistically significant. Because unknown confounders could exist, further prospective studies are needed to identify possible causation.
